# Time to normalise protected characteristics in written assessments: A mixed methods study

**DOI:** 10.12688/mep.19877.1

**Published:** 2024-03-08

**Authors:** Adam Shepherd, Sam Bott, Laila Abdullah, Russell Hearn

**Affiliations:** 1 GKT School of Medical Education, King's College London, London, SE1 1UL, UK

**Keywords:** Single best answer question, assessment, diversity, cultural competency, demographic, curriculum, LGBT, ethnicity

## Abstract

**Background:**

Despite increasing endeavours to incorporate teaching material on healthcare for minority groups into medical school curricula, including cultural competency, there is a lack of research exploring medical students' comprehension of this. With age and gender as the only demographic information routinely provided in undergraduate single best answer (SBA) questions, the diversity of patients encountered by doctors in clinical practice is not fairly represented in assessments. This study examined the impact of not declaring gender or explicitly indicating LGBT+ identities and ethnicity, on how medical students evaluate clinical scenarios through SBA questions.

**Methods:**

200 medical students across clinical years completed 15 SBA questions in an online simulated exam. Participants were randomised to control and test groups testing different types of patient demographic information in question stems.

**Results:**

Linear regression modelling demonstrated overall statistically nonsignificant differences between groups. The largest effect size was seen in the LGBT+ question intervention group, which had the fewest white and postgraduate participants. Older and more senior medical students performed better generally. White participants overall significantly outperformed non-white participants; this difference was eliminated when answering a mix of question styles. Using a mix of question styles produced statistically significant differences, with participants scoring worse on LGBT+ and ethnicity style questions.

**Conclusion:**

Increased depth and breadth of clinical experience enables medical students to approach clinical scenarios with more flexibility. Unfamiliarity with minority patient groups may have impacted their performance in this study. For medical education to remain contemporary in preparing future clinicians to interact with diverse patient groups, assessments need to normalise the presence of these patients.

## Introduction

Medical school graduates must understand how diversity in patient populations affects behaviors and outcomes in healthcare settings ( GMC, 2015). Endeavors to incorporate this into medical school curricula have cultivated cultural competency teaching, yet this often remains ad hoc without a framework to integrate it longitudinally into the wider curriculum ( Bentley *et al.*, 2008; Dubin *et al.*, 2018). Furthermore, there are currently no formal assessment standards to examine whether medical students have achieved the required understanding of how patient diversity impacts healthcare access and delivery.

### Diversity in medicine

Diversity broadly refers to the spectrum and variety of social characteristics and the ethnic backgrounds of individuals. While there is an increasing recognition of minority groups within healthcare settings, the lesbian, gay, bisexual, transgender and queer (LGBTQ+) community continues to face disproportionate health inequalities and discrimination ( Bachmann & Gooch, 2018; Government Equalities Office, 2018; Moll *et al.*, 2019; Nama *et al.*, 2017). Ethnic minorities also experience great health disparities, driven in part by the implicit biases of healthcare providers ( Cormack *et al.*, 2018; Greenaway *et al.*, 2020; Hamed *et al.*, 2022).

Exposure to a broad range of patient groups during medical school significantly increases students’ readiness and confidence in dealing appropriately and sensitively with these patients in the future ( Nowaskie & Patel, 2020). Saha *et al.* (2008) found that medical graduates from universities with ethnically diverse student bodies perceived themselves as being more able to care for ethnic minority patients. While research indicates that medical students generally have favorable attitudes towards minority groups, a lack of explicit curriculum content on LGBTQ+ health care leaves them feeling unconfident about caring for these patients ( Greene *et al.*, 2018; Parameshwaran *et al.*, 2017).

Cultural competence is the ability to work effectively with patients from a variety of social groups and backgrounds to ensure that the delivery of care aligns with their needs ( Nair & Adetayo, 2019). Within medical education, this approach predominantly focuses on the delivery of teaching within the curriculum. As cultural competence exists at the individual, interpersonal, and institutional levels, merely modifying the taught components is inadequate to produce a syllabus that is fit for practice ( Li *et al.*, 2023). A collaborative effort between learners, teachers, and organizations is required to develop a culturally competent curriculum ( McGregor *et al.*, 2019) to subsequently achieve the outcome of a culturally competent healthcare workforce and system.

The purpose of procuring culturally competent doctors is to bridge disparities in healthcare delivery when there is a lack of doctor-patient concordance. Ethnic minority patients lack ethnic concordance and face greater challenges in accessing the same standard of care as their counterparts, including receiving the correct treatment in a timely manner ( Anderson *et al.*, 2020; McGregor *et al.*, 2019).

Therefore, the issue of diversity in medicine is related to both patients and doctors. There are ongoing calls to increase the diversity of the medical profession through recruitment, tackling differential attainment, and career progression support ( Butler *et al.*, 2019; Ellis *et al.*, 2023; GMC, 2019a; Orom *et al.*, 2013; Saha *et al.*, 2000; Woolf *et al.*, 2011). This needs to be considered when examining the medical school curriculum to strengthen equity in education.

### Assessments of the curriculum

Gaps remain with respect to the inclusion of minority groups in undergraduate medical education. Although international efforts are underway to incorporate more material about the LGBTQ+ community, ethnic minorities, and their healthcare needs into medical school curricula ( Cooper *et al.*, 2018; Green *et al.*, 2022; Micheal & Marjadi, 2018; Salkind *et al.*, 2019), teaching on cultural competency is still largely overlooked and deemed a low priority ( Nair & Adetayo, 2019). Developments in assessments are similarly lacking.

In moving towards more inclusive syllabuses, medical schools are ‘decolonizing’ their curricula by evaluating not only what is taught, but how it came to be taught, by whom it is taught, and what assessment practices are utilized ( Hartland & Larkai, 2020; Lokugamage *et al.*, 2020; Mbaki *et al.*, 2021). The six-step approach to curriculum development requires evaluation components to determine whether an educational intervention achieves the desired outcome ( Thomas *et al.*, 2015). Therefore, assessments are an integral part of undergraduate medical education. These compound written, practical, and portfolio elements ( Epstein, 2007). For medical education to remain contemporary in preparing future doctors for interacting with diverse patient groups, amendments have to be made not only to teaching components, but also to subsequent assessments.

It is widely accepted that assessments align with curriculum. While the Association of American Medical Colleges has developed a Tool for Assessing Cultural Competence Training, it is a blueprint for what taught components should entail ( Jernigan *et al.*, 2016). It contains suggestions on where to include practical exams, reflective practice, and communication skill building within a curriculum but does not formally assess students’ learned abilities and competences.

In the UK, the Medical Licensing Assessment (MLA) content map similarly highlights that medical students must behave in accordance with equality and diversity principles as a key area within clinical and professional capabilities to be assessed through the MLA ( GMC, 2019b). At present there is limited peer reviewed research on the assessment of cultural competency ( Dogra *et al.*, 2016), with none exploring the impact of including diverse patient demographic factors in single best answer (SBA) questions on exam performance. SBA multiple choice questions are a reliable examination method and comparatively simple to produce ( Al-Wardy, 2010).

Current style standards for SBA questions set out that stems denote patient age and gender, i.e. “a 60 year old man” ( MSCAA, 2018). This presumes that patients are cisgender with no reference to other demographic information. Ethnicity and sexuality are typically only included where clinically relevant, to minimize the potential stereotyping of minority groups. However, this emphasis on minority differences can reinforce rather than dismantle stereotyped perceptions ( Zanting *et al.*, 2020). An alternative approach to normalizing the existence of diversity would be to present diverse patients in everyday clinical contexts. In this study, we explored how SBA question style affects medical students’ evaluation and interpretation of clinical scenarios based on the level of patient demographic information.

## Methods

This was a mixed-methods study where participants were blinded to the test group. The quantitative performance of participants answering simulated SBA questions and their qualitative experience of doing so were assessed. Before the simulated exam, participants were informed that this study evaluated SBA styles, but blinded them to assess the impact of patient demographic information within question stems. This study was conducted using the Qualtrics survey platform in June 2022. After signing a written consent form, Qualtrics randomized the participants into control and test groups; the authors were blinded as to which participant was allocated to which group. After participation, the subjects were debriefed and informed of the aim of the study. Ethical approval was granted by the BDM Research Ethics Subcommittee on 16/05/2022 (ref: HR/DP-21/22-28747).

### Study design

All the groups received 15 corresponding SBA questions in the same order, varying only in the level of patient demographic details provided. Additional demographic information was not designed to be relevant to the clinical picture presented. We adopted a positivist approach, in that all questions were intended to have the same single correct answer, regardless of the patient described in the stem. The simulated SBA questions were written by the first two authors and reviewed for clinical accuracy by the third author, who is a GP with expertise in medical education and assessments.

The control group questions were formulated in the current style standard, denoting the patient's age and gender (“A 60 year old man”). Test groups 1–3 were neutral (“A 60 year old person”); indicated LGBT+ identities (“A 60 year old gay man”); and noted ethnicity (“A 60 year old Chinese man”), respectively. The fourth test group consisted of a randomized selection of questions from all the groups. To reflect the modern vernacular, terminology regarding demographic information was based on the 2021 UK census ( ONS, 2021).

Participants were instructed to spend approximately 18 min on the simulated exam questions, proportional to the amount of time they would receive in a real exam to answer 15 SBA questions. The questions covered the medical curriculum content of the first two clinical years of King’s College London. After the simulated exam, participants answered Likert-scale questions about their perception of answering SBA questions.

### Participants

Medical students in their clinical years were invited to participate. The invitations were disseminated via student networks. Demographic characteristics of the 200 participants are presented in Table 1. Gender, sexuality, and ethnicity questions comprised open response boxes, enabling participants to self-determine how they identify. Participants were offered a £5 gift voucher, and received answers and explanations for the SBA questions to aid their learning.

**Table 1.** Participant Demographics (%).

	Control Group	Neutral TG	LGBTQ+ TG	Ethnicity TG	Mixed TG
Male	47.5	52.5	45	60.5	52.5
Female	52.5	47.5	55	39.5	47.5
Heterosexual	65.9	67.5	55	73.7	68.3
Gay	17.1	15	25	10.5	12.2
Lesbian	12.2	12.5	15	15.8	12.2
Bisexual	2.4	2.5	–	–	7.3
Asexual	–	–	2.5	–	–
Not sure	2.4	–	2.5	–	–
Prefer not to say	–	2.5	–	–	–
White	80.5	84.6	73	89.6	82.5
African/Caribbean	2.4	–	–	2.6	–
South Asian	17.1	7.7	8.1	2.6	10
East Asian	–	2.6	8.1	2.6	5
Mixed	–	5.1	10.8	2.6	2.5
<20 years	12.2	5	7.5	10.5	12.2
21–25 years	56.1	60	55	44.7	48.8
26–30 years	17.1	25	22.5	18.5	19.5
31–35 years	4.9	5	7.5	10.5	7.3
36–40 years	7.3	5	5	13.2	9.8
>41 years	2.4	–	2.5	2.6	2.4
Year 2	24.4	17.5	25	21.2	19.5
Year 3	56.1	55	57.5	57.8	63.4
Year 4	14.6	22.5	12.5	15.8	9.8
Year 5	4.9	5	5	5.2	7.3
Previous University Education	85	73	68	84	78
No Previous University Education	15	28	33	16	22

### Analysis

Regression modelling was performed using Stata 17.0, with a p value of <0.05, demonstrating statistical significance. To facilitate statistical analysis, participants’ ethnicity and sexuality were grouped as white and non-white, and LGB+ and heterosexual, respectively, to achieve more balanced sample sizes. This provided recognition of participants’ diversity during data collection while retaining the usefulness of data for analysis ( Vivienne *et al.*, 2023). The average rating was determined for the post-test Likert scale questions.

## Results

**Figure 1.**
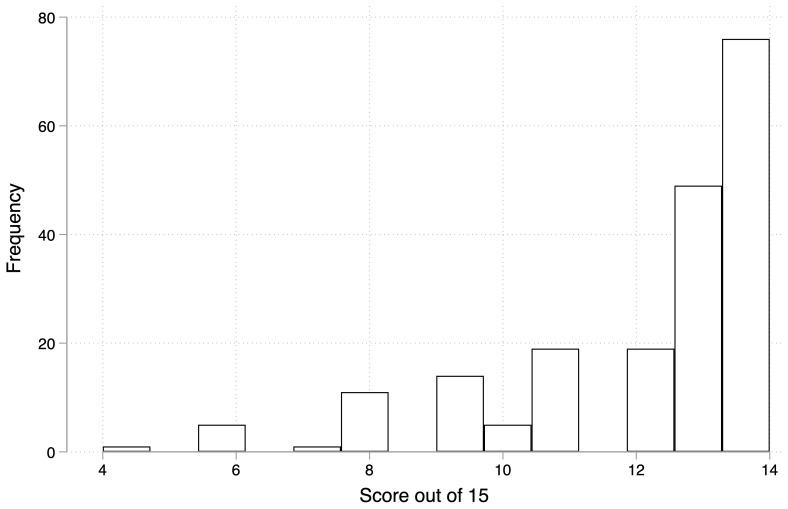
Overall score distribution of all participants.

The score distribution of the participants is shown in Figure 1. Approximately 75% of the participants answered at least of the 12/15 questions correctly. 

### Overall performance

**Figure 2.**
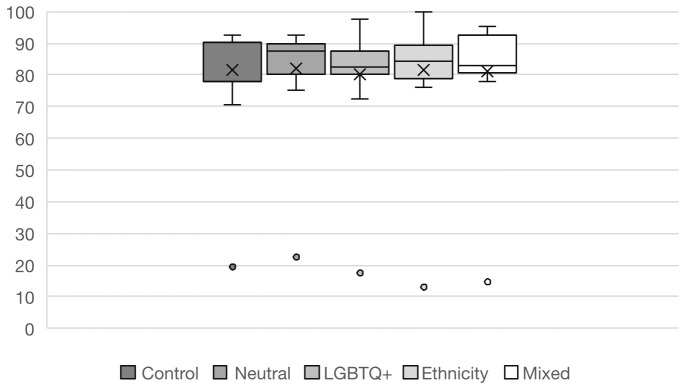
Overall question performance in percentage by group. Boxes depict interquartile range with lines representing median, and crosses mean values. Bars define maximum and minimum percent of correct answers. Dots are outliers.

The analysis of question performance is shown in Figure 2. Overall, there was broadly similar performance between the control and test groups. All the groups performed substantially worse on one question. This was designated as an outlier and removed from further statistical analysis because flawed SBA questions are less predictive of students’ true performance ( Omer *et al.*, 2016).

A simple linear regression comparing overall performance between groups was statistically insignificant (R ^2^ = 0.002, F(4, 195) = 0.12, p = 0.976), indicating that group allocation did not explain the variance in exam scores (see Table 2). The largest effect size was observed when comparing the LGBT+ test group (TG) to the Control Group (β = -0.248, p = 0.646).

**Table 2.** Simple linear regression of exam scores by group.

	β Coefficient	Std error	p value	95% Confidence intervals [Lower limit, upper limit]
Neutral TG	-0.048	0.539	0.930	-1.1101, 1.0149
LGBT+ TG	-0.248	0.539	0.646	-1.3101, 0.8149
Ethnicity TG	0.034	0.546	0.950	-1.0426, 1.1106
Mixed TG	-0.073	0.536	0.891	-1.1291, 0.9828

### Sub-analysis of overall performance

Multiple linear regression for group and participant characteristics is reported in Table 3, with the overall regression model being statistically significant (R ^2^ = 0.46, F(13, 179) = 11.74, p = 0.000). 

**Table 3.** Multiple linear regression of exam scores by group and participant characteristics.

	β Coefficient	Std error	p value	95% Confidence intervals [Lower limit, upper limit]
Neutral TG	-0.006	0.431	0.99	-0.855, 0.844
LGBT+ TG	-0.249	0.435	0.568	-1.1067, 0.6098
Ethnicity TG	-0.263	0.419	0.53	-1.0889, 0.5627
Mixed TG	-0.251	0.409	0.54	-1.0592, 0.5563
Year 3	1.035	0.474	0.03	0.1004, 1.9695
Year 4 & 5	-0.511	0.535	0.341	-1.5674, 0.5453
Postgraduate	0.392	0.456	0.391	-0.5084, 1.2925
21–25 years	0.313	0.683	0.647	-1.0344, 1.6602
26–30 years	0.877	0.81	0.28	-0.7216, 2.4758
≥31 years	0.816	0.828	0.325	-0.8171, 2.4496
Male	0.649	0.323	0.046	0.01036, 1.2868
LGB+	0.589	0.326	0.073	-0.0546, 1.2333
White	1.926	0.496	0.000	0.9478, 2.9048

Performance in the test groups remained statistically insignificant compared to that in the control group. Controlling for participant characteristics, the negative effect of Ethnicity TG and Mixed TG increased modestly (Ethnicity TG β = -0.034 to β = -0.263, p = 0.53; Mixed TG β = -0.073 to β = -0.251, p = 0.54).

***Year of study*.** Participants in Year 3 achieved significantly higher scores than those in Year 2 (β = 1.035, p = 0.03). Students in years 4 and 5 performed moderately worse than Year 2 students, but this was statistically insignificant (β = -0.511, p = 0.341). 

***Previous university education*.** Postgraduate students achieved a modestly higher score than students whose first degree was in medicine (β = 0.392, p = 0.391).

***Age*.** An increasing positive, statistically insignificant effect size was observed up to age 30 compared with participants aged ≤20 (21–25 years β = 0.313, p = 0.647; 26–30 years β = 0.877, p = 0.28; ≥31 years β = 0.816, p = 0.325). 

***Gender*.** Overall, male participants achieved a significantly higher score than did female participants (β = 0.649, p = 0.046). 

***Sexuality*.** Across the groups, LGB+ participants performed better than self-described heterosexual participants, but this was not statistically significant (β = 0.589, p = 0.073). 

***Ethnicity*.** There was a significant difference in the overall performance between white and non-white participants, with white participants scoring markedly higher (β = 1.926, p = 0.000).

### Mixed test group sub-analysis

A simple linear regression comparing the overall performance between question styles was statistically significant (R ^2^ = 0.207, F(3, 160) = 0.13.9, p = 0.000) ( Table 4). Overall, participants performed significantly worse with LGBT+ and Ethnicity style questions (LGBT+ style β Coefficient = -0.683, p = 0.000; Ethnicity style β Coefficient = -0.781, p = 0.000) with minimal differences in neutral style questions compared to the control style.

**Table 4.** Simple linear regression of exam scores by style.

	β Coefficient	Std error	p value	95% Confidence intervals [Lower limit, upper limit]
Neutral style	0.122	0.175	0.488	-0.224, 0.468
LGBT+ style	-0.683	0.175	0.000	-1.029, -0.337
Ethnicity style	-0.781	0.175	0.000	-1.127, -0.434

Multiple linear regressions for style and participant characteristics are reported in Table 5; the overall regression model was statistically significant (R ^2^ = 0.471, F(12, 151) = 11.19, p = 0.000), with the same effect sizes observed comparing performance in the test styles to control style when controlling for participant characteristics.

**Table 5.** Multiple linear regression of exam scores by style and participant characteristics.

	β Coefficient	Std error	p value	95% Confidence intervals [Lower limit, upper limit]
Neutral Style	0.122	0.147	0.409	-0.1692, 0.4131
LGBT+ Style	-0.683	0.147	0.000	-0.9741, -0.3918
Ethnicity Style	-0.781	0.147	0.000	-1.0717, -0.4893
Year 3	0.545	0.272	0.047	0.0076, 1.0826
Year 4 & 5	0.033	0.301	0.914	-0.5619, 0.6274
Postgraduate	0.616	0.2404	0.011	0.1414, 1.0913
21–25 years	0.059	0.2545	0.815	-0.4432, 0.5625
26–30 years	0.134	0.3135	0.670	-0.4857, 0.7532
≥31 years	0.091	0.3182	0.776	-0.5381, 0.7193
Male	0.195	0.1357	0.153	-0.0734, 0.4629
LGB+	-0.0009	0.1495	0.995	-0.2963, 0.2946
White	-0.182	0.2313	0.433	-0.6389, 0.2749

***Gender*.** Male participants performed marginally better than did female participants (β coefficient = 0.195, p = 0.153). 

***Age*.** A statistically insignificant increase in exam score was seen across age bands up to age 30 (21–25 years β Coefficient = 0.059, p = 0.815; 26–30 years β Coefficient = 0.134, p = 0.670).

***Sexuality*.** No significant differences were observed in performance between heterosexual and LGB+ participants.

***Ethnicity*.** Non-white participants performed marginally better overall than white participants, but this was statistically insignificant (β coefficient -0.182, p=0.433).

***Previous university education*.** Postgraduate students performed significantly better than students with no prior higher education (β coefficient = 0.616, p = 0.011).

***Year of study*.** Year 3 students performed significantly better than Year 2 students (β Coefficient = 0.545, p = 0.047) with minimal difference observed for Year 4 and 5 participants (β Coefficient = 0.033, p = 0.914). 

### Post-test questions

**Figure 3.**
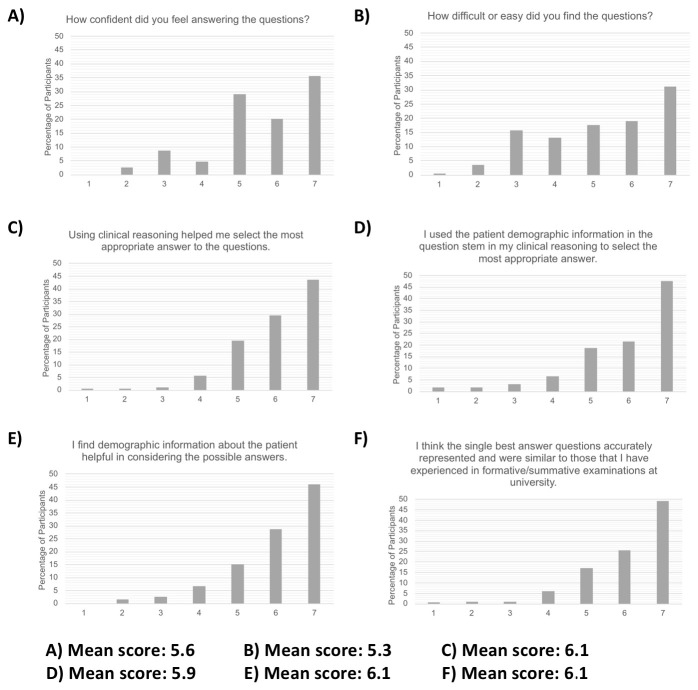
Post-test Likert scale questions, overall score distribution of all participants and mean scores.

Overall, participants felt that the simulated SBA questions accurately reflected the real SBA exam questions that they have had as part of formal assessments at medical school (mean score 6.1/7, see Figure 3). Participants felt fairly confident in answering the simulated SBA questions (mean score 5.6/7) and the questions were regarded as moderately easy (mean score 5.3/7). The majority of the participants perceived themselves to have used clinical reasoning in answering the questions (mean score of 6.1/7). Similarly, they felt that they had utilized the patient demographic information provided (mean score of 5.9/7). The majority perceived patient demographic information to be detail which added value to the question stem (mean score 6.1/7).

Although the post-test questions revealed that participants perceived themselves to utilize patient demographic information to aid in answering SBA questions, in practice, the simulated SBA results showed no significant difference with the addition of the patient's sexuality or ethnicity in the question stem or neutral questions. With a mixture of question styles, LGBT+ and ethnicity-style questions significantly affected how participants answered questions.

## Discussion

The purpose of assessments in medical education is to provide a valid and reliable method for evaluating students’ competence ( Van der Vleuten, 1996). Doctors and medical students come into contact with a diverse range of patients. Therefore, their clinical practice must be tailored to understand the importance of utilizing demographic information for patient care. Although the teaching curriculum in medical education is ever-changing and rapidly becoming diversified ( Salkind *et al.*, 2019), it has not transcended undergraduate assessment to the same extent.

Beagan (2003) found that the introduction of a longitudinal module on cross-cultural health in a Canadian medical school had a limited impact on students’ perceived understanding of cultural competency issues, indicating the need to evaluate the effectiveness of education initiatives.

In a review of cultural competency interventions in medical schools, 65% used non-standardized evaluations such as surveys, which do not formally assess the long-term retention of knowledge and skills ( Deliz *et al.*, 2020). While a national review of LGBTQ+ healthcare-related content in UK medical schools reported that 44% of responding institutions used multiple-choice questions as part of their assessment for this curricular component, it did not specify the nature or context of how these are constructed or what specifically they examine ( Tollemache *et al.*, 2021). Analyses of the inclusion of ethnicity as a demographic identifier in question banks for the United States Medical Licensing Examination did not extend to evaluating the impact of ethnicity on question performance ( Erickson *et al.*, 2023; Ripp & Braun, 2017).

Currently, there is no peer-reviewed literature examining the effects of diversifying SBA questions to align with real-life clinical practice and how this affects students’ performance. In this study, we explored whether providing more or less details about patient demographics affects how medical students interpret and solve clinical problems posed by SBA questions.

### Formulating fair assessments

Where statistically significant differences in question performance were identified, they were largely attributable to the participant characteristics. Unsurprisingly, participants who were older and in the more senior years of their degree performed better. Of all male participants, 55.9% were over 26 years of age, and 93.1% had completed a previous university degree. This compares to 16.6% of female participants aged 26 or above, and 60.4% holding a prior degree. 

Academic underperformance among non-white medical students and doctors is an ongoing problem ( Jones *et al.*, 2021; Vaughan *et al.*, 2015; Woolf *et al.*, 2011). In this study, 44.6% of white participants were above the age of 26, and 88% reported having previously studied at university versus 6% of non-white participants in the same age category and 28.6% with previous university education. While participants in the LGBT+ TG group had the largest difference in scores compared with the control group, this group had the least number of white and postgraduate participants. These factors may have contributed to the difference in performance observed in this study, although with uneven participant numbers, inferences must be made with care.

Participants were least likely to perform differently on neutral questions than in the control group. Hanckel and Shepherd (2023) questioned the usefulness of gender as a proxy for anatomy and physiology in medical contexts. Omitting this demographic information may eliminate potential implicit biases and enable medical students to focus on the clinical details provided. The additional demographic information provided in other question styles may have adversely affected the participants’ interpretation of the clinical scenarios because of their lack of familiarity with minority patient groups. 

Correlating the cognitive level of assessments to the cognitive level of the students being assessed aids in developing more compelling evaluations of their performance ( Thompson & Griffin, 2021). The use of demographic identifiers, or lack thereof, in SBA questions may therefore be helpful to introduce in the later years of undergraduate medicine to more closely resemble the real-life contexts that medical students are exposed to, and provide an opportunity for them to demonstrate their adaptability to different circumstances.

The more context a question includes, the more it tests the application of knowledge, as students need to evaluate each piece of information for its relevance to the case presented ( Schuwirth & van der Vleuten, 2004). Care needs to be taken not to disadvantage neurodiverse medical students, whose academic performance can be adversely affected by increasing cognitive load ( Clouder *et al.*, 2020), although the impact of this specifically on SBA questions has not yet been explored. Furthermore, the inclusion of additional demographic information must not impede the comprehensibility of exam questions to avoid disadvantaging students who take exams in a foreign language ( Verbree *et al.*, 2023).

A Universal Design for Learning approache has been promoted to redress differential attainment. By utilizing a variety of assessment methods, students can demonstrate their strengths ( Fyfe *et al.*, 2022; Luke, 2021; Odukoya *et al.*, 2021). As part of developing an inclusive curriculum, this may help challenge systemic barriers affecting learning opportunities and outcomes for medical students. Moreover, a side effect of increasing diversity in the undergraduate curriculum is that minority group students feel less marginalized within the medical profession ( Luke, 2021).

### Incorporating diversity in assessments

The static nature of SBA questions has prevented them from being considered effective, non-stigmatizing measures of cultural competence ( Dogra *et al.*, 2016). However, rather than testing students’ recall of fixed knowledge about being culturally competent, SBA questions should aim to incorporate cultural competency as part of generating holistic clinical reasoning processes.

Other assessment methods have shortcomings. Although cultural competency knowledge can be examined through essays, it does not test the clinical application of knowledge ( Betancourt, 2003; Dogra *et al.*, 2016). Portfolio reflections and case-based discussions are frank approaches to appraise attitudes and knowledge, yet they often lack standardized marking.

While practical exams are more flexible in the content that is assessed, cultural competencies should be actively integrated across these exams rather than being designed within isolated stations ( Dogra *et al.*, 2016; Patrício *et al.*, 2013). Simulation-based learning is easily adaptable. However, as with practical exams, this requires the availability of minority group actors and patients who are willing to be exposed to possible cultural incompetence from inexperienced students.

Such a minority tax also befalls minority group clinicians, faculty members, and medical students ( Fyfe *et al.*, 2022; Obedin-Maliver *et al.*, 2011). Tollemache *et al.* (2021) found that UK medical schools often rely on these individuals and charities to deliver cultural competency education. This can be problematic if they do not have the pedagogic knowledge and skills needed to formulate curriculum content, which risks reinforcing biases and stigmas ( Kai *et al.*, 2001; Muntinga *et al.*, 2020).

Overall, a multimodal approach to assessing cultural competency, including written, practical, and portfolio elements, is pivotal for producing more holistic clinicians. It has been shown that medical students focus their learning on what they perceive to be assessed on ( Newble & Jaeger, 1983). Actively including diverse patient demographic information in SBA question stems will prompt medical students to learn and have an awareness of a diverse patient population, thereby visibilising this often ‘hidden’ curriculum.

### Normalising diversity in medicine

The inclusion of demographic information, such as ethnicity and sexuality, in medical education has been contentious with concerns about propagating pigeonholing of minority groups ( Lee *et al.*, 2022; Nawaz & Brett, 2009). In analyses of third-party practice question banks for the United States Medical Licensing Exam, it has been found that questions detailing patient ethnicity tend to do so in stigmatizing ways by accentuating racial tropes and stereotypes ( Erickson *et al.*, 2023; Ripp & Braun, 2017). However, not including minority groups in medical school assessments risks signifying that patients’ sociocultural backgrounds and experiences are irrelevant.

Zanting *et al.* (2020) caution against framing cultural identifiers in simplistic ways, as this risks reinforcing stereotypes, thereby exoticizing minority groups. Although only referencing ethnicity or sexuality in SBA questions when it is clinically relevant has been done with the aim of reducing stigmatization, when the standard is to discuss patients in neutral terms this practice highlights differences in minority groups and can work to position them as deviant (eg “a 50 year old man” versus “a 50 year old black man”). Erasing the natural diversity of patient populations in fear of stereotyping also results in harm, through ill health in minority groups being overinflated or underrecognized due to a lack of familiarity from doctors ( Lokugamage *et al.*, 2020).

Although Zanting *et al.* (2020) suggest that conceptualizing demographic information as fixed and absolute enhances stereotyping by ignoring the dynamic nature of culture, in clinical practice, some identifiers are unchanging. While gender identity can be an evolving process of becoming, it can also be a fully developed state of the self ( Hanckel & Shepherd, 2023). To broaden diverse patient representation, Erickson *et al.* (2023) call for exam questions “ *to be more patient centered rather than patient labeling*”.

### Strengths and limitations

A limitation of this preliminary study is the small sample size for some participant characteristics. Therefore, caution is required when extrapolating the findings presented here. Additionally, the lack of previously published work in this area poses a challenge in contextualizing the results. However, the overall sample size of this study makes the general findings more robust. 

Although the online survey platform improved accessibility, it limited the monitoring of participants to mimic exam conditions. Advertising this study via student groups likely targeted more proactive and engaged medical students, who may already be academically stronger than their peers, which may have skewed the results.

Future research in this area should ideally simulate exam conditions more closely with a larger sample of SBA questions, monitoring participants, and more diverse recruitment methods to attract a wider range of participants. The inclusion of neurodiversity as a participant characteristic would further help to ensure that SBA styles are formatted without prejudice.

## Conclusion

This research contributes to the development of best practices for writing SBA questions in undergraduate medical education. Currently, there is a single style standard for SBA questions, and using a mixture of styles may be beneficial in testing clinical reasoning, cultural competence, and scientific knowledge more refinedly. Care should be taken to ensure that medical students are not unduly disadvantaged by the style of the exam question.

It may be that the lack of confidence in the medical profession when caring for minority patients partly emanates from the curation of assessment material, resulting in predominantly binary-type formats.

Denying diversity in undergraduate assessments propagates a disservice to patients and colleagues. A multimodal approach is needed to effectively assess cultural competency in undergraduate medical students to confirm that they attain the outcomes expected of medical graduates ( GMC, 2015). SBA questions can form part of this by normalizing the presence of diversity in medicine and positioning minority group patients as active agents seeking and accessing health care. Assessment formulation should be the responsibility of appropriately trained personnel.

Overall, the aim is for cultural competence and diversity to be integrated into medical school curricula and assessment, instead of being separated as a standalone component. Normalizing the use of more diverse demographic information within SBA questions without amplifying the stereotyping of minority groups will ultimately contribute towards preparing medical students for the wider experience they will be part of as doctors leading towards better patient care. 

## Data Availability

King's College London Data Repository: Diversity representation in SBAQs raw data nonidentifiable, https://doi.org/10.18742/24468220 ( Shepherd *et al.*, 2023). This project contains the following underlying data: -   one spreadsheet containing the participant characteristics and simulated exam responses. The underlying data are available under the terms of the King’s Data Access Agreement. It is not openly available due to conditions of participant consent and may be shared on request with academic or clinical researchers for non-commercial research on completion of a data access agreement. To request access, please email the address noted at the top of the record, including in your email the name and DOI of the dataset. King's College London Data Repository: Diversity representation in SBAQs question sets, https://doi.org/10.18742/25067918.v1 ( Shepherd *et al.*, 2024) This project contains the following extended data: SBA questions for all four groups Likert post-test questions Data are available under the terms of the Attribution-NonCommercial 4.0 International (CC-BY NC 4.0).
