# Time to normalise protected characteristics in written assessments: A pilot study

**DOI:** 10.12688/mep.19877.2

**Published:** 2026-04-24

**Authors:** Adam Shepherd, Sam Bott, Laila Abdullah, Russell Hearn

**Affiliations:** 1 GKT School of Medical Education, King's College London, London, SE1 1UL, UK

**Keywords:** Single best answer question, assessment, diversity, cultural competency, demographic, curriculum, LGBT, ethnicity

## Abstract

**Background:**

With increasing endeavours to incorporate teaching material on healthcare for underrepresented groups into medical school curricula, there remains a lack of research exploring the integration of this into assessments. With age and gender as the only demographic information routinely provided in single best answer (SBA) questions, this does not represent the diversity of patients encountered by doctors in clinical practice. This pilot study assessed how representation of patient demographic characteristics influenced medical students answering SBA questions.

**Methods:**

200 medical students in their clinical years completed 15 SBA questions in an online simulated exam. Participants were randomised to control and test groups. The control group denoted age and gender, with test groups including neutral stems denoting age only; stems denoting LGBTQ+ identities; stems denoting ethnicity; and a mix of stems. Post-exam Likert scale questions explored their experience of the simulated exam.

**Results:**

Linear regression modelling demonstrated overall statistically nonsignificant differences between groups, indicating that assignment to control or test group does not explain differences in exam performance. Using a mix of question stems produced statistically significant differences, with participants scoring worse on question stems including LGBT+ identities and ethnicity.

**Conclusion:**

As a pilot study these results suggest diversification of SBA questions does not inherently disadvantage students. A multimodal approach is needed to integrate diversity into medical school curricula and assessments. SBA questions can form part of this by mirroring diverse patient groups encountered in real-life clinical practice and positioning them as active agents seeking and accessing health care.

## Introduction

Medical school graduates must understand how diversity in patient populations affects behaviors and outcomes in healthcare settings ( GMC, 2015). Although equality, diversity, and inclusion (EDI) has been cultivated in undergraduate medical education teaching, there are currently no formal assessment standards to examine whether medical students have achieved the required understanding of how patient diversity impacts healthcare access and delivery.

### Diversity in medicine

In this study, we refer to diversity as the spectrum and variety of personal characteristics and backgrounds of individuals. Diversity is often under-represented in medicine. This means some population groups are often not proportionally represented relative to the general population ( Smart & Harrison, 2017). Individuals from, for example, racially minoritised groups and the LGBTQ+ community continue to experience health inequities and discrimination ( Bachmann & Gooch, 2018; Cormack *et al*., 2018; Government Equalities Office, 2018; Greenaway *et al*., 2020; Hamed *et al*., 2022; Moll *et al.*, 2019; Nama *et al.*, 2017). This is in part driven by the implicit biases of healthcare providers ( Cormack *et al.*, 2018; Greenaway *et al.*, 2020; Hamed *et al.*, 2022). Training and education of medical students is key in reducing these health disparities because exposure to a broad range of patient groups has been shown to increase students’ readiness and confidence in caring for patients from diverse backgrounds in the future ( Greene *et al.*, 2018; Michael & Marjadie, 2018; Nowaskie & Patel, 2020; Parameshwaran *et al.*, 2017; Salking *et al*., 2019). Saha *et al.* (2008) found that medical graduates from universities with ethnically diverse student bodies perceived themselves as being more able to care for racially minoritised patients.

In medical education, EDI teaching is often a stand-alone component rather than integrated into wider learning experiences, despite this being largely unsuccessful with regard to long-term knowledge retention ( Atkinson *et al*., 2022; Bentley *et al*., 2008; Dubin *et al*., 2018; Heidari *et al*., 2024; Henry *et al*., 2024). Heidari *et al*. (2024) also highlight that doctors who come from diverse backgrounds have an innate understanding of cultural nuances beyond what can be taught in a classroom. Patients who have ethnic concordance with their doctors have been shown to face fewer challenges in accessing care, including receiving the correct treatment in a timely manner ( Anderson *et al.*, 2020; McGregor *et al.*, 2019).

Therefore, the issue of diversity in medicine is related to both patients and doctors. There are ongoing calls to increase the diversity of the medical profession through recruitment, tackling differential attainment, and career progression support ( Butler *et al.*, 2019; Ellis *et al.*, 2023; GMC, 2019a; Orom *et al.*, 2013; Saha *et al.*, 2000; Woolf *et al.*, 2011).

### Assessments of the curriculum

The purpose of assessments in medical education is to provide a valid and reliable method for evaluating students’ competence ( Van der Vleuten, 1996). Assessments are an integral part of undergraduate medical education and it is widely accepted that assessments align with the curriculum.

In the UK, the Medical Licensing Assessment (MLA) content map highlights that medical students must behave in accordance with equality and diversity principles as a key area within clinical and professional capabilities to be assessed through the MLA ( GMC, 2019b). The Association of American Medical Colleges developed a Tool for Assessing Cultural Competence Training which includes suggestions on how to incorporate this in practical exams, reflective practice, and communication skill building ( Jernigan *et al*., 2016). Yet at present there is limited peer reviewed research on the assessment of diversity ( Dogra *et al.*, 2016).

In a review of teaching initiatives in medical schools about health disparities for minoritised patient groups, 64.9% used non-standardised evaluations such as surveys which do not formally assess the long-term retention of knowledge and skills ( Deliz *et al*., 2020). A national review of LGBTQ+ healthcare-related content in UK medical schools reported that 44% of responding institutions used multiple-choice questions, but the nature or context of these questions was not specified ( Tollemache *et al*., 2021). Analyses of the inclusion of ethnicity as a patient characteristic in question banks for the United States Medical Licensing Examination did not extend to evaluating the impact of ethnicity on question performance ( Erickson *et al*., 2023; Ripp & Braun, 2017).

Caution has been raised that static representations of personal characteristics in assessments can cause harm by stereotyping under-represented groups ( Dogra *et al*., 2016). Rather than testing students’ recall of fixed knowledge of diversity, assessments should aim to incorporate EDI as part of holistic clinical reasoning processes.

Current style standards for SBA questions set out that stems denote patient age and gender, i.e. “a 60 year old man” ( MSCAA, 2018). This presumes that patients are cisgender with no reference to other demographic information. Single best answer questions in particular have been critiqued for emphasising minority differences and thereby reinforcing rather than dismantling stereotyped perceptions ( Dogra *et al*., 2016; Zanting *et al*., 2020). SBA multiple choice questions are, however, a reliable examination method and comparatively simple to produce ( Al-Wardy, 2010). An alternative approach to normalising diversity in SBA questions is to present diverse patients in everyday clinical contexts.

In this study, we explored representing diverse patient characteristics in SBA question stems affects how medical students answer them.

## Methods

This was a pilot study using a double blind, randomised experimental intervention design, to assess whether the inclusion of diverse personal characteristic information in SBA question stems affected medical students’ performance on a simulated SBA exam. It was conducted using the Qualtrics survey platform.

### Single best answer question development

The simulated SBA questions were written by the first two authors and reviewed for clinical accuracy by the third author, who is a GP with expertise in medical education and assessments. See Shepherd *et al*. (2024) for the data repository information to access the full question set.

**Figure 1.**
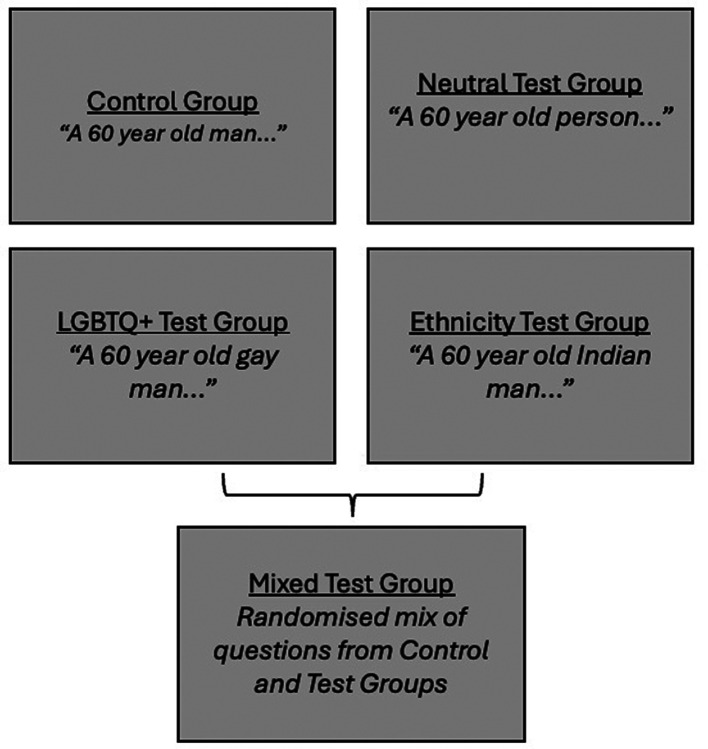
Control and test group question stems.

Question stems differed in the type of personal characteristics provided about the patient in the scenario, as outlined in Figure 1 below. Ethnicity, gender, and sexual orientation were chosen as these are protected characteristics under the UK Equality Act 2010, providing anti-discrimination protections. To reflect the modern vernacular, terminology regarding demographic characteristics was based on the 2021 UK census ( ONS, 2021). The patient demographic characteristics were designed to not alter the clinical scenario.

We adopted a positivist approach with each question designed to have one correct answer, regardless of the protected characteristics described in the stem. Questions were planned to be of equivalent difficulty and covered the medical curriculum content of the first two clinical years.

Our hypothesis was that medical students would interpret the question stems differently because of the patient demographic characteristics provided.

### Participants

Medical students in their clinical years were invited to participate. Invitations were disseminated via student networks. The participant information sheet and consent form was integrated into the Qualtrics survey, requiring students to read and sign these before participating. This provided the opportunity to return later if they needed time to consider their choice to participate.

Participants were asked about their own demographic characteristics of age, ethnicity, gender, religion, sexuality, year of study, and previous university degrees.

Before the simulated exam, participants were informed that this study evaluated SBA question styles, however participants were blind to variation in patient characteristics within question stems being the focus of this study.

### Intervention

After signing a written consent form, 200 participants were randomly allocated by the Qualtrics system into one of five study arms, control group and four test groups. Authors were also blinded as to participant group allocation. Figure 1 shows examples of demographic characteristics in question stems for each group.

A neutral test group was included to assess if the absence of patient characteristics affected students’ interpretation of clinical scenarios through removing implicit biases.

All the groups received 15 corresponding SBA questions in the same order, varying only in the patient demographic characteristic details provided. Participants were instructed to spend approximately 18 minutes on the simulated exam questions, proportional to the amount of time they would receive in a real exam to answer 15 SBA questions.

After the simulated exam, participants answered Likert-scale questions about their perception of answering the SBA questions. This was for quality control purposes to evaluate if the SBA questions were comparable to real exam questions and to ascertain participants’ perceptions of the relevancy of patient demographic characteristics. After participation, the subjects were debriefed and informed of the aim of this study. Data collection took place in June 2022. Participants received £5 gift vouchers and explanations for the SBA questions to aid their learning. Ethical approval was granted by the BDM Research Ethics Subcommittee on 16/05/2022 (ref: HR/DP-21/22–28747).

### Analysis

Regression modelling was performed using Stata 17.0, with a p value of <0.05 demonstrating statistical significance.

## Results

Participant demographic characteristics of the 200 participants are presented in Table 1. Gender, sexual orientation, and ethnicity questions comprised open response boxes, enabling participants to self-determine how they describe themselves. Where participants provided answers that did not match the demographic questions, these were excluded.

**Table 1.** Participant characteristics by number of participants (%).

	Control Group	Neutral TG	LGBTQ+ TG	Ethnicity TG	Mixed TG
Male	19 (47.5)	21 (52.5)	18 (45)	23 (60.5)	21 (52.5)
Female	21 (52.5)	19 (47.5)	22 (55)	15 (39.5)	19 (47.5)
Heterosexual	27 (65.9)	27 (67.5)	22 (55)	28 (73.7)	28 (68.3)
Gay	7 (17.1)	6 (15)	10 (25)	4 (10.5)	5 (12.2)
Lesbian	5 (12.2)	5 (12.5)	6 (15)	6 (15.8)	5 (12.2)
Bisexual	1 (2.4)	1 (2.5)	–	–	3 (7.3)
Asexual	–	–	1 (2.5)	–	–
Not sure	1 (2.4)	–	1 (2.5)	–	–
Prefer not to say	–	1 (2.5)	–	–	–
White	33 (80.5)	33 (84.6)	27 (73)	34 (89.6)	33 (82.5)
African/Caribbean	1 (2.4)	–	–	1 (2.6)	–
South Asian	7 (17.1)	3 (7.7)	3 (8.1)	1 (2.6)	4 (10)
East Asian	–	1 (2.6)	3 (8.1)	3 (2.6)	2 (5)
Mixed	–	2 (5.1)	4 (10.8)	1 (2.6)	1 (2.5)
<20 years	5 (12.2)	2 (5)	3 (7.5)	4 (10.5)	5 (12.2)
21–25 years	23 (56.1)	24 (60)	22 (55)	17 (44.7)	20 (48.8)
26–30 years	7 (17.1)	10 (25)	9 (22.5)	7 (18.5)	8 (19.5)
31–35 years	2 (4.9)	2 (5)	3 (7.5)	4 (10.5)	3 (7.3)
36–40 years	3 (7.3)	2 (5)	2 (5)	5 (13.2)	4 (9.8)
>41 years	1 (2.4)	–	1 (2.5)	1 (2.6)	1 (2.4)
Year 2	10 (24.4)	7 (17.5)	10 (25)	8 (21.2)	5 (19.5)
Year 3	23 (56.1)	22 (55)	23 (57.5)	22 (57.8)	20 (63.4)
Year 4	6 (14.6)	9 (22.5)	5 (12.5)	6 (15.8)	8 (9.8)
Year 5	2 (4.9)	2 (5)	2 (5)	2 (5.2)	3 (7.3)
Previous University Education	35 (85)	29 (73)	27 (68)	32 (84)	32 (78)
No Previous University Education	6 (15)	11 (28)	13 (33)	6 (16)	9 (22)

The majority of participants were white (80%), aged 21–25 years (53%), and in their third year of medical school (58%). Most participants had a prior university degree (77.5%). Notably, the LGBTQ+ test group had the highest number of LGB+ participants (45% vs 26–36% in other groups) and racially minoritised participants (27% vs 10–19.5%).

**Figure 2.**
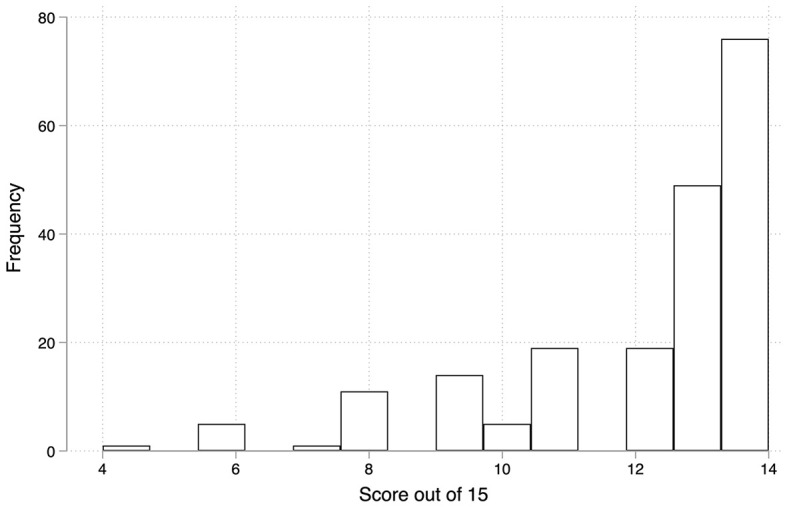
Overall score distribution of all participants.

The score distribution of the participants is shown in Figure 2. Approximately 75% of the participants answered at least of the 12/15 questions correctly.

### Overall performance

**Figure 3.**
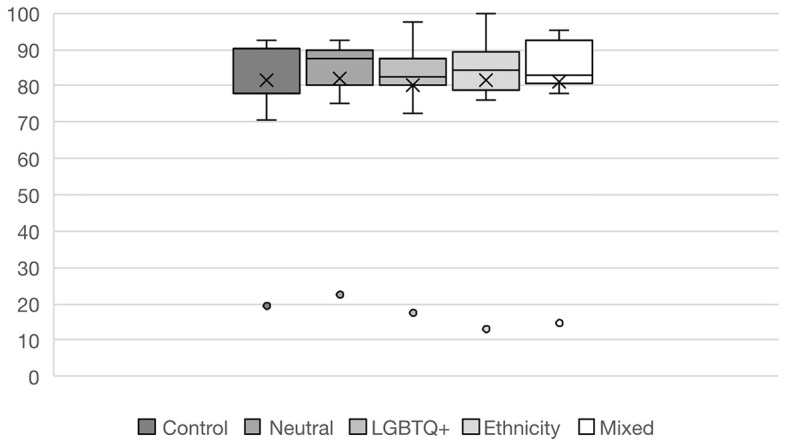
Overall question performance in percentage by group. Boxes depict interquartile range with lines representing median, and crosses mean values. Bars define maximum and minimum percent of correct answers. Dots are outliers.

The analysis of question performance is shown in Figure 3. Overall, there was broadly similar performance between the control and test groups. All the groups performed substantially worse on one question. This was designated as an outlier and removed from further statistical analysis because flawed SBA questions are less predictive of students’ true performance ( Omer *et al.*, 2016).

A simple linear regression comparing overall performance between groups was statistically insignificant (R ^2^ = 0.002, F(4, 195) = 0.12, p = 0.976), indicating that group allocation did not explain the variance in exam scores (see Table 2). The largest effect size was observed when comparing the LGBT+ test group (TG) to the Control Group (β = −0.248, p = 0.646).

**Table 2.** Simple linear regression of exam scores by group.

	β Coefficient	Std error	p value	95% Confidence intervals [Lower limit, upper limit]
Neutral TG	−0.048	0.539	0.930	−1.1101, 1.0149
LGBT+ TG	−0.248	0.539	0.646	−1.3101, 0.8149
Ethnicity TG	0.034	0.546	0.950	−1.0426, 1.1106
Mixed TG	−0.073	0.536	0.891	−1.1291, 0.9828

### Mixed test group sub-analysis

20.5% of participants were randomised to the Mixed TG. A simple linear regression comparing the overall performance between question stems was statistically significant (R ^2^ = 0.207, F(3, 160) = 0.13.9, p = 0.000) ( Table 3). Overall, participants performed significantly worse with question stems including LGBT+ identities and Ethnicity (LGBT+ stems β Coefficient = −0.683, p = 0.000; Ethnicity stems β Coefficient = −0.781, p = 0.000) with minimal differences in neutral questions compared to the control stems.

**Table 3.** Simple linear regression of exam scores by stem.

	β Coefficient	Std error	p value	95% Confidence intervals [Lower limit, upper limit]
Neutral stems	0.122	0.175	0.488	−0.224, 0.468
LGBT+ stems	−0.683	0.175	0.000	−1.029, −0.337
Ethnicity stems	−0.781	0.175	0.000	−1.127, −0.434

### Post-test questions

**Figure 4.**
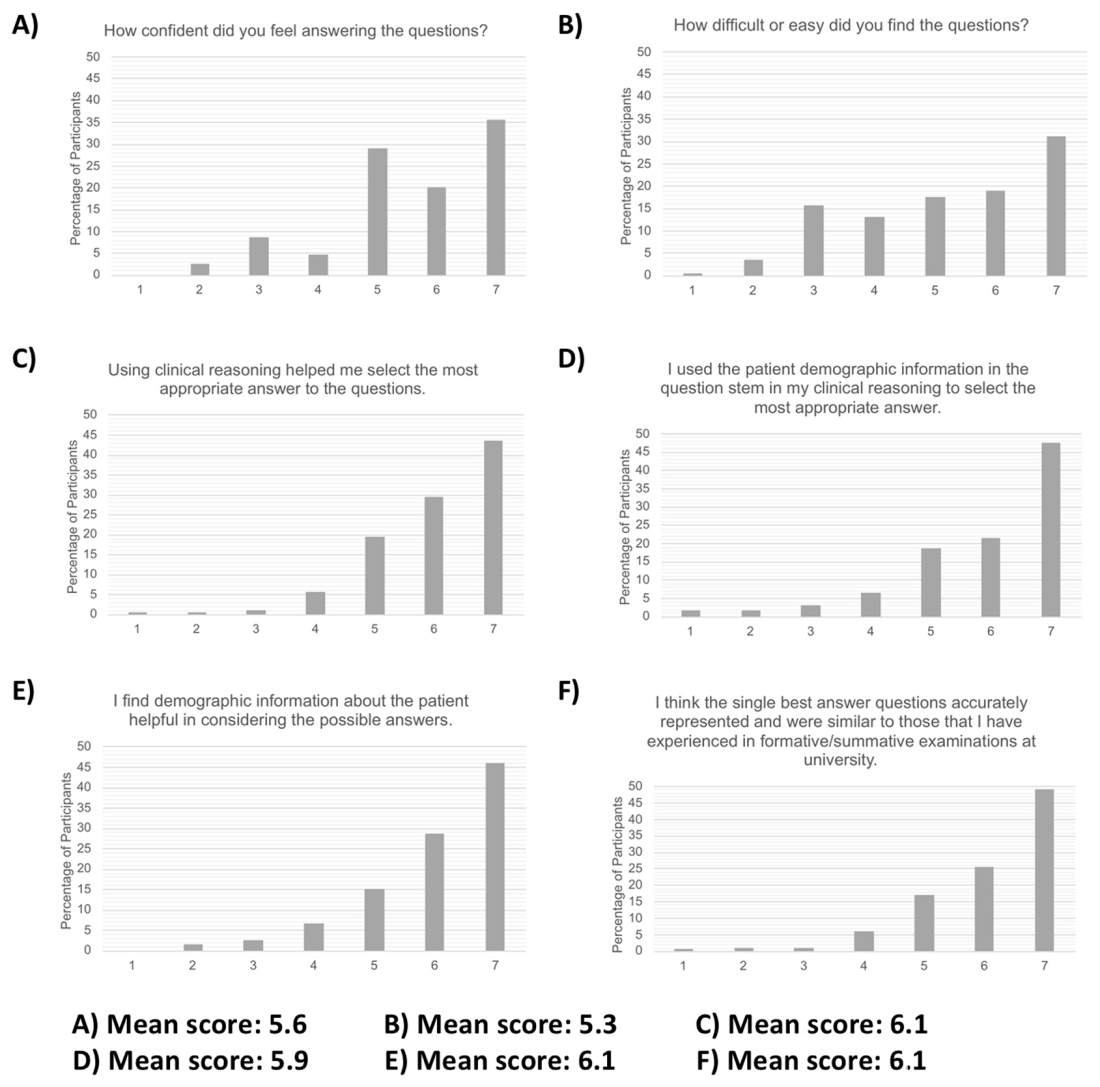
Post-test Likert scale questions, overall score distribution of all participants and mean scores.

Overall, participants felt that the simulated SBA questions accurately reflected the real SBA exam questions that they have had as part of formal assessments at medical school (mean score 6.1/7, see Figure 4). Participants felt fairly confident in answering the simulated SBA questions (mean score 5.6/7) and the questions were regarded as moderately easy (mean score 5.3/7). The majority of the participants perceived themselves to have used clinical reasoning in answering the questions (mean score of 6.1/7). Similarly, they felt that they had utilised the patient demographic information provided (mean score of 5.9/7). The majority perceived patient demographic information to be detail which added value to the question stem (mean score 6.1/7).

Although the post-test questions revealed that participants perceived themselves to utilise patient demographic information to aid in answering SBA questions, in practice, the simulated SBA results did not conclusively show a significant difference with the addition of the LGBT+ identities or ethnicity in the question stem or neutral questions.

## Discussion

In this pilot study we examined the responses to SBA questions depending on patient characteristics in the question stem. The simple linear regression model analysing overall exam performance was statistically insignificant: Allocation to control or test groups did not explain the difference in exam scores observed between participants.

Analysing the Mixed TG overall scores, simple linear regression modelling demonstrated that participants performed worse on questions with ethnicity and LGBT+ patient characteristics. The more context a question includes, the more it tests the application of knowledge, as students need to evaluate each piece of information for its relevance to the case presented ( Schuwirth & van der Vleuten, 2004). Participants in the Mixed TG were least likely to perform differently between the control and neutral stems. Hanckel and Shepherd (2023) questioned the usefulness of gender as a proxy for anatomy and physiology in medical contexts given the complex nature of human biology. Omitting this characteristic may eliminate potential implicit biases and enable medical students to focus on the clinical details provided. The additional demographic characteristics provided in the LGBT+ and ethnicity question stems may therefore have adversely affected the participants’ interpretation of the clinical scenarios because of their lack of familiarity with these patient groups. A larger research trial with more SBA questions is warranted to investigate the nature in which ethnicity and LGBT+ identities may influence clinical reasoning.

Correlating the cognitive level of assessments to the cognitive level of the students being assessed aids in developing more compelling evaluations of their performance ( Thompson & Griffin, 2021). The use of demographic characteristics, or lack thereof, in SBA question stems may helpful to introduce in the later years of undergraduate medicine degrees to more closely resemble the real-life contexts that medical students are exposed to, and provide an opportunity for them to demonstrate their adaptability to different circumstances.

Yet care needs to be taken not to disadvantage neurodiverse medical students, whose academic performance can be adversely affected by increasing cognitive load ( Clouder *et al.*, 2020), although the impact of this specifically on SBA questions has not yet been explored. Furthermore, the inclusion of patient characteristics must not impede the comprehensibility of exam questions to avoid disadvantaging students who take exams in a foreign language ( Verbree *et al.*, 2023).

The inclusion of demographic characteristics, such as ethnicity and sexual orientation, in medical education has been contentious with concerns about propagating pigeonholing of minoritised groups ( Lee *et al.*, 2022; Nawaz & Brett, 2009). Zanting *et al*. (2020) caution against framing cultural identifiers in simplistic ways, as this risks reinforcing stereotypes, thereby exoticising minoritised groups. Only referencing ethnicity or sexual orientation in SBA questions when it is clinically relevant has been done with the aim of reducing stigmatisation, yet when the standard is to discuss patients in neutral terms this practice highlights differences in minoritised groups and can work to position them as deviant.

In analyses of third-party practice question banks for the United States Medical Licensing Exam, it was found that questions detailing patient ethnicity tend to do so in stigmatising ways by accentuating racial tropes and stereotypes ( Erickson *et al.*, 2023; Ripp & Braun, 2017). However, erasing the natural diversity of patient populations in fear of stereotyping also results in harm, through ill health in minoritised groups being overinflated or underrecognised due to a lack of familiarity from doctors ( Lokugamage *et al*., 2020). It moreover risks signifying that patients’ sociocultural backgrounds and experiences are irrelevant.

Although Zanting *et al*. (2020) suggest that conceptualising patient demographic characteristics as fixed and absolute enhances stereotyping by ignoring the dynamic nature of culture, in clinical practice, some characteristics are unchanging. For example, while gender identity can be an evolving process of becoming, it can also be a fully developed state of the self ( Hanckel & Shepherd, 2023). To broaden diverse patient representation, Erickson *et al*. (2023) call for exam questions “to be more patient centered rather than patient labeling”. In this pilot study we took an unequivocal approach in how patient characteristics were provided in the SBA question stems to assess the direct impact of this on participants’ exam performance. Interestingly, although the majority of participants in the post-test questions reported utilising the patient characteristic information provided in answering the SBA questions, this did not appear to definitively affect their exam performance as the overall simple linear regression model was non-significant. Future research is needed to explore the best approach for introducing diversity into SBA questions.

Medical students have been shown to focus their learning on what they perceive to be assessed on ( Newble & Jaeger, 1983). Actively normalising the inclusion of diverse patient characteristics in assessments will therefore prompt students to learn and have an awareness of a diverse patient population. Assessments in medical education compound written, practical, and portfolio elements ( Epstein, 2007). Although this study focused on SBA questions, different assessment formats have different utilities. Essays provide space for deeper exploration of a topic but often do not test the application of clinical knowledge. While portfolio reflections and case based discussions are opportunities to focus in individual learning experiences, these often lack standardised marking ( Betancourt, 2003; Dogra *et al*., 2016). Practical exams are more flexible in the content of the assessment, but EDI should be actively integrated across these exams rather than being designed within isolated stations ( Dogra *et al*., 2016; Patrício *et al*., 2013). Simulation-based learning is easily adaptable but, as with practical exams, requires the availability of minoritised group actors and patients who are willing to be exposed to possible offence from inexperienced students. This minority tax of relying on minoritised group clinicians, faculty members, students, and charities to deliver EDI education must be considered when designing curricula and curricular assessments ( Fyfe *et al*., 2022; Obedin-Maliver *et al*., 2011; Tollemache *et al*., 2021). This can be problematic if they do not have the pedagogic knowledge and skills needed to formulate curriculum content, which risks reinforcing biases and stigmas ( Kai *et al*., 2001; Muntinga *et al*., 2020).

A Universal Design for Learning approach has been promoted to redress differential attainment: By utilising a variety of assessment methods including written, practical, and portfolio elements students can demonstrate their varying strengths ( Fyfe *et al.*, 2022; Luke, 2021; Odukoya *et al.*, 2021). A similar multimodal strategy should be adopted to integrate EDI in assessments throughout medical education.

Interestingly, of all white participants in this study, 88% reported having previously studied at university versus 28.6% of racially minoritised participants. Academic underperformance among racially minoritised medical students and doctors is an ongoing problem affecting undergraduate and postgraduate medical education ( Jones *et al.*, 2021; Vaughan *et al.*, 2015; Woolf *et al.*, 2011). Developing an inclusive curriculum may help challenge systemic barriers affecting learning opportunities and outcomes for medical students through feeling less marginalised within the medical profession ( Luke, 2021).

### Strengths and limitations

A limitation of this pilot study is the small sample size for some participant characteristics. Therefore, caution is required when extrapolating the findings presented here. The lack of previously published work in this area also poses a challenge in contextualising the results. However, the overall sample size of this study makes the general findings more robust. Additionally, students perceived the SBA questions to be an accurate representation of real-life written exams they have undertaken at their medical school.

Although the online survey platform improved accessibility, it limited the monitoring of participants to mimic exam conditions. Advertising this study via student groups likely targeted more proactive and engaged medical students, who may already be academically stronger than their peers, which may have skewed the results.

Future research in this area should ideally simulate exam conditions more closely with a larger sample of SBA questions with a broader range of question difficulty, monitoring participants, and more diverse recruitment methods to attract a wider range of participants. The inclusion of neurodiversity as a participant characteristic would further help to ensure that SBA stems are formatted without prejudice.

## Conclusion

This research contributes to the development of best practices for writing SBA questions in undergraduate medical education. As a pilot study it demonstrates that widening representation of diverse patient characteristics within SBA questions to reflect the general population does not inevitably have an adverse effect on exam performance.

Denying diversity in undergraduate assessments propagates a disservice to patients and medical professionals within current and future workforces. A multimodal approach is needed to effectively integrate diversity into medical school curricula, instead of being separated as standalone components. For medical education to remain contemporary in preparing future doctors for interacting with diverse patient groups, amendments have to be made not only to teaching material, but also to subsequent assessments to ensure that medical graduates attain expected outcomes in understanding how EDI interacts with healthcare ( GMC, 2015). Normalising the use of more diverse demographic information in assessments without amplifying the stereotyping of minoritised groups can contribute towards preparing medical students for the wider experience they will be part of as doctors leading towards better patient care. SBA questions can form part of this by positioning minoritised patient groups as active agents seeking and accessing health care.

## Data availability

### Underlying data

King’s College London Data Repository: Diversity representation in SBAQs raw data nonidentifiable, https://doi.org/10.18742/24468220 ( Shepherd *et al.*, 2023).

This project contains the following underlying data:

- one spreadsheet containing the participant characteristics and simulated exam responses.

The underlying data are available under the terms of the King’s Data Access Agreement. It is not openly available due to conditions of participant consent and may be shared on request with academic or clinical researchers for non-commercial research on completion of a data access agreement. To request access, please email the address noted at the top of the record, including in your email the name and DOI of the dataset.

### Extended data

King’s College London Data Repository: Diversity representation in SBAQs question sets, https://doi.org/10.18742/25067918.v1 ( Shepherd *et al.*, 2024).

This project contains the following extended data:

• SBA questions for all four groups
• Likert post-test questions

Data are available under the terms of the Attribution-NonCommercial 4.0 International (CC-BY NC 4.0).
