# Supplementary material for: Time to normalise protected characteristics in written assessments: A pilot study
Source: MedEdPublish (2016). 2026 Apr 24;14:10. Originally published 2024 Mar 8. [Version 2] doi: 10.12688/mep.19877.2 (PMC13197748; doi:10.12688/mep.19877.2)
Supplement: Peer review history

# Reviewer report: Reviewer response for version 2

**DOI:** 10.21956/mep.23127.r45793

**Published:** 2026-05-23

**Authors:** Sara Reza

**Affiliations:** 1 Quaid-e-Azam Medical College, Bahawalpur, Pakistan

The revised version of the manuscript is materially improved. The authors have removed the “mixed methods” approach to the article and retitled it as a pilot study, clarified the test/control groups in the abstract, included the data-collection timeframe, and described the randomization, blinding, the Qualtrics procedure, the Likert questions and the rationale for the different versions of the SBA stems. The paper now has a clearer and more defensible scope than the earlier version.

 However, the revision fully resolves the main methodological and interpretive concerns raised in the previous reviews. There still seems to be an overemphasis on statistically limited and potentially inconsistent findings and the statistical method used still lacks adequate justification for the design of the study.

 The central result remains a non-significant between-group comparison: the authors report R² = 0.002, F (4,195) = 0.12, p = 0.976, meaning that group allocation explained essentially none of the variance in overall exam scores. Despite this, the conclusion still states that the findings “demonstrate” that widening representation “does not inevitably have an adverse effect.” This is too strong. A non-significant result in a pilot study does not show absence of effect, particularly without a power calculation or an equivalence/non-inferiority framework.

 The mixed-test-group sub-analysis is also difficult to interpret. The paper reports statistically worse performance on LGBTQ+ and ethnicity stems within the mixed group, but appears to analyze question-stem effects using simple linear regression. This does not seem to account for repeated responses nested within participants and questions, or for item difficulty. A mixed-effects logistic model, or a similar item-level model, would seem more appropriate. The authors should also make clear which analysis was primary and which analyses were secondary or exploratory. In particular, they should state whether the mixed-test-group sub-analysis was pre-specified or conducted post hoc, as this affects how much weight should be placed on that finding. The reported statistic “F (3,160) = 0.13.9” on page 7 also appears to contain a typographical or reporting error.

 The study also depends on the assumption that the only meaningful difference between the different SBA stem versions was the demographic wording. At present, the manuscript does not provide enough evidence to support that assumption. The authors should give more detail on how the SBA items and their parallel stem versions were reviewed or piloted to ensure they were equivalent in difficulty, clarity, and clinical relevance. For example, it would be helpful to know whether item-writing guidance was used, whether there was expert review beyond clinical accuracy, whether any piloting was undertaken, or whether item-level performance indices were examined.

 Confounding and participant imbalance are still not adequately handled. The table of participant characteristics shows some imbalance across groups; for example, the LGBTQ+ test group had the highest proportion of LGB+ participants and racially minoritized participants. The authors mention these differences, but they do not report adjusted models including year of study, prior degree, ethnicity, sexuality, gender, or other relevant participant characteristics. Because participants’ own demographics are collected and reported, the authors need to explain more clearly whether these variables were intended as descriptive context, covariates, or exploratory subgroup variables. At present, their inclusion still risks distracting from the main research question.

 The revised paper is better framed as a pilot study, but some claims remain stronger than the findings support. In particular, the statement that the study “contributes to the development of best practices” still feels overstated unless the authors make clear that this contribution is preliminary and hypothesis-generating. Similarly, causal or overly reassuring claims, such as “does not disadvantage students”, should be replaced with more cautious pilot-study wording.

 The discussion is less rambling than the earlier version, but it still goes beyond what the data can directly support. Sections on neurodiversity, minority tax, Universal Design for Learning, differential attainment, and wider curricular reform are relevant to the broader topic, but not all are clearly linked to the findings of this study. These points would be more persuasive if they were shortened and framed more explicitly as implications for future research or future assessment design, rather than as conclusions arising from the present data.

**Suggested required revisions before approval**

1. Add a power/sample-size statement, even if post hoc or explicitly framed as pilot feasibility.
2. Reanalyze item-level performance using an appropriate model that accounts for participant-level and item-level clustering.
3. Clarify whether participant demographics were collected for descriptive, covariate, or subgroup purposes.
4. Add adjusted analyses or explain why adjustment is not appropriate.
5. Replace causal or reassuring claims such as “does not disadvantage students” with cautious pilot-study wording.
6. Shorten the discussion and move broader curricular implications into a clearly labelled “future research/implications” paragraph.
7. Correct statistical reporting errors, especially the mixed-group F statistic and the use of “p = 0.000,” which should be reported as “p < 0.001.”

Overall, this is a worthwhile and timely pilot study, and the authors have clearly responded to several earlier criticisms. However, the paper should not be approved in its current form. The manuscript could become acceptable after major revisions, particularly if the analysis is strengthened and the conclusions are made more cautious.

Have any limitations of the research been acknowledged?

Partly

Is the work clearly and accurately presented and does it cite the current literature?

Partly

If applicable, is the statistical analysis and its interpretation appropriate?

No

Are all the source data underlying the results available to ensure full reproducibility?

Partly

Is the study design appropriate and is the work technically sound?

Partly

Are the conclusions drawn adequately supported by the results?

No

Are sufficient details of methods and analysis provided to allow replication by others?

Partly

Reviewer Expertise:

NA

I confirm that I have read this submission and believe that I have an appropriate level of expertise to confirm that it is of an acceptable scientific standard, however I have significant reservations, as outlined above.

# Reviewer report: Reviewer response for version 2

**DOI:** 10.21956/mep.23127.r45795

**Published:** 2026-05-22

**Authors:** Barbara Jennings

**Affiliations:** 1 University of East Anglia, Norwich, England, UK

I did not peer review version 1 of the paper and have focused attention on how thoroughly the authors have responded to initial reviewer comments (which I agreed with) in V2 of their manuscript. I notice that one change has been to reframe the article to describe a *pilot study* and with a focus on quantitative outcomes.

*Scope, Ethics, and Presentation*

 This article fits within the field of medical education and addresses the important topic of including protected demographic characteristics into simulated patient scenarios for assessment. Institutional review board approval, most data availability and author contributions are transparent; and a second version of the article has been written in response to peer review.

*Strengths of Article*

 This addresses a clear gap in the medical education literature. The central importance of integrating and embedding diversity in all aspects of a medical curriculum (and assessment in particular) is well articulated throughout.

*Problems to address for version 2*

 The authors have not thoroughly addressed all of the careful suggestions made by the V1 reviewers or explained/justified their amendments in sufficient detail in the section between the abstract and introduction.

 Introduction:

- In particular, more attention is needed with clarity of arguments in the Introduction. By condensing this, the authors are lumping together citations from diverse articles without enough detail for me to understand the exact point being made, or which piece of evidence (from a citation) is presented.

Methods:

- Methods and data transparency: There should be direct repository information for SBAs to readers embedded within this manuscript – not via a link to another citation (Shepherd 2024).
- I note your hypothesis in the Methods section “that medical students would interpret the stems differently….” But the authors need to demonstrate understanding of testing the null hypothesis. As the previous reviewer explained there is a risk of an under powered study here and some consideration of sample size and the notional effect size (of the variables tested) should be explored in more detail. This point also applies to the Discussion and Strengths/Limitations section in particular. This type of exploration would be reasonable for a pilot study.
- Linked to the above, can the authors justify the use of simple linear regression?

Results Interpretation/ Discussion:

- The authors conclude a lack of significance in linear regression analysis (i.e. no evidence to reject a null hypothesis) and yet they unpick and suggest reasons for numerical differences between groups/ variables in the discussion.

The aspirations and ideas behind this article are strong and the authors share concepts and important underpinning literature. The statistical analysis and methodology limitations should be reviewed. This could be coupled with rigorous engagement with (response to) the initial peer review reports.

Have any limitations of the research been acknowledged?

Partly

Is the work clearly and accurately presented and does it cite the current literature?

Partly

If applicable, is the statistical analysis and its interpretation appropriate?

I cannot comment. A qualified statistician is required.

Are all the source data underlying the results available to ensure full reproducibility?

Yes

Is the study design appropriate and is the work technically sound?

Partly

Are the conclusions drawn adequately supported by the results?

Partly

Are sufficient details of methods and analysis provided to allow replication by others?

Partly

Reviewer Expertise:

The reviewer, Barbara Jennings, has no conflicts of interest to declare. I have expertise in faculty development, widening access to medical careers, and quantitative research. I did not peer review version 1 of the paper and have focused attention on how thoroughly the authors have responded to initial reviewer comments (which I agreed with) in V2 of their manuscript.

I confirm that I have read this submission and believe that I have an appropriate level of expertise to confirm that it is of an acceptable scientific standard, however I have significant reservations, as outlined above.

# Reviewer report: Reviewer response for version 2

**DOI:** 10.21956/mep.23127.r45730

**Published:** 2026-05-22

**Authors:** Rhoda Meyer

**Affiliations:** 1 Stellenbosch University, Cape Town, South Africa

**Abstract**

 The abstract is much more succinct, reflecting a clearer aim and a more detailed methodology.

**Introduction**

 The concept of diversity and how it relates to this study is well explained. The background section appears to be more streamlined. However, a clear definition of cultural competence would strengthen this section.

 The authors introduce the concepts of diversity and cultural competence and the need for this in medical education. However, the link between the two has not been explicated, creating an assumption that the two are directly related.

**Methods**

 Although this section has been revised, it would be good if the authors could indicate that this is a quantitative study

 It is still not clear whether participants were informed that their demographics/characteristics would be linked to their performance **.**

**Conclusion**

 Although the authors state that the study contributes to the development of best practices for writing SBAs, this level of contribution is not clear in their subsequent statements.

Have any limitations of the research been acknowledged?

Yes

Is the work clearly and accurately presented and does it cite the current literature?

Partly

If applicable, is the statistical analysis and its interpretation appropriate?

I cannot comment. A qualified statistician is required.

Are all the source data underlying the results available to ensure full reproducibility?

Partly

Is the study design appropriate and is the work technically sound?

Partly

Are the conclusions drawn adequately supported by the results?

Partly

Are sufficient details of methods and analysis provided to allow replication by others?

No

Reviewer Expertise:

NA

I confirm that I have read this submission and believe that I have an appropriate level of expertise to confirm that it is of an acceptable scientific standard, however I have significant reservations, as outlined above.

# Reviewer report: Reviewer response for version 1

**DOI:** 10.21956/mep.21293.r41018

**Published:** 2025-01-30

**Authors:** Rhoda Meyer

**Affiliations:** 1 Stellenbosch University, Cape Town, South Africa

Thank you for the opportunity to review this article. Overall, I found the article to be interesting. There is no conflict of interest. The study addresses a gap in medical education by exploring how SBA question style affects medical students’ evaluation and interpretation of clinical scenarios based on the level of patient demographic information. 

**Abstract**

- The abstract provides a good overview of the article but would benefit from revision in terms of aspects related to the methodology.
- The authors state that gender is one of the demographic types of information routinely included in SBA’s but examines the impact of not declaring gender in SBAs – this needs to be clarified.
- The nature of the demographic variations between the control and test groups needs to be included.
- The significance of linking student’s own demographics to performance is not clear since the aim of the study was to examine the impact of not declaring gender or LGBT+ identities and ethnicity.
- To understand what the authors are arguing in terms of the clinical experience and how students approach clinical scenarios, the level of clinical experience of the participants should have been included.

**Introduction**

- The authors introduce the concepts of diversity and cultural competence and the need for this in medical education. However the link between the two has not been explicated, creating an assumption that the two are directly related.
- In addition, cultural competence is much more than ‘being able to work effectively with patients from a variety of social groups and backgrounds’, and this needs to be problematised.
- The literature used to justify statements related to the lack of a framework to integrate cultural competence longitudinally into the wider curriculum is outdated. More recent literature needs to be sought and cited to support claims, e.g. Atkinson et al. 2022.
- The authors argue that a collaborative effort is needed to develop a culturally competent curriculum. However these statements contradict other arguments offered by the authors such as ‘modifying the taught components is inadequate…’ Furthermore, it is not clear how this study contributes to a more collaborative effort.
- Another example of an incoherent argument is the statement on increasing diversity through recruitment etc. and then considering this when examining the curriculum.
- The authors mention a six-step approach to curriculum development, but it is not clear what this approach is and how it is linked to the study, if at all.
- It is also indicated that assessments are an ‘integral part of undergraduate medical education’. While this is true, it is not clear what the authors are arguing. Perhaps if the six-step approach is further unpacked, the role of assessment will be revealed.
- While assessments are an integral part of learning, it is not clear why the authors chose to focus on assessments rather than teaching and learning. A strong justification is needed.
- How the authors controlled for question difficulty across different versions is unclear.
- There is also no indication of the timeframe of the study.

**Methods**

- The methods section requires a fair amount of work. It is not clear why a mixed method approach was selected.
- While the quantitative aspect of the approach is described, there is minimal detail on the qualitative aspect.
- A Likert scale was used but it is not clear is this formed part of the quantitative aspect of the study.
- It is not clear why the demographics of the students were important for this study and how it contributed to the aim.
- **Furthermore the authors need to indicate whether participants were informed that their demographics/characteristics  will be linked to their performance.**
- Information about how the researchers controlled for potential confounding variables is lacking.
- Data analysis for the qualitative aspect is not included in the article.

**Discussion**

- The introductory section of the discussion provides information that is more relevant to the introduction and background.
- At times, the discussion section focuses on the demographics of the students rather than answering the research question.
- Certain aspects in the discussion do not relate to the findings, e.g. the relevance of neurodiverse medical students for this study is not clear.
- Certain statements require further unpacking. For example, the authors state that ‘actively including diverse patient demographic information in SBA’ leads to ‘visibilising’ the hidden curriculum. The link between the two needs clarity.
- This section requires extensive revision.

**Conclusion **

- Although the authors state that the study contributes to the development of best practices for writing SBAs, this level of contribution is not clear in their subsequent statements.

Overall, this article requires major revision.

Have any limitations of the research been acknowledged?

Yes

Is the work clearly and accurately presented and does it cite the current literature?

Partly

If applicable, is the statistical analysis and its interpretation appropriate?

I cannot comment. A qualified statistician is required.

Are all the source data underlying the results available to ensure full reproducibility?

Partly

Is the study design appropriate and is the work technically sound?

Partly

Are the conclusions drawn adequately supported by the results?

Partly

Are sufficient details of methods and analysis provided to allow replication by others?

No

Reviewer Expertise:

Health Professions Education

I confirm that I have read this submission and believe that I have an appropriate level of expertise to state that I do not consider it to be of an acceptable scientific standard, for reasons outlined above.

# Reviewer report: Reviewer response for version 1

**DOI:** 10.21956/mep.21293.r39330

**Published:** 2024-12-10

**Authors:** Kevin McConville

**Affiliations:** 1 University of Dundee, Dundee, Scotland, UK

This research positions itself as mixed methods; however, it draws only on Likehert scales and quantitative analysis. Therefore, this is not true of the title, i.e. no qualitative approaches are noted.

 The messages derived from this work, given that the study appears ? underpowered and statistically non-significant, making any interpretation difficult to ascertain.

 The discussions are wide-ranging and rambling, and I do not see what, if any, added value is provided in the results. The authors have tried to make a paper out of a set of non-statistical significant results.

 I would not recommend indexing at this time.

Have any limitations of the research been acknowledged?

Partly

Is the work clearly and accurately presented and does it cite the current literature?

Partly

If applicable, is the statistical analysis and its interpretation appropriate?

I cannot comment. A qualified statistician is required.

Are all the source data underlying the results available to ensure full reproducibility?

Partly

Is the study design appropriate and is the work technically sound?

No

Are the conclusions drawn adequately supported by the results?

No

Are sufficient details of methods and analysis provided to allow replication by others?

Partly

Reviewer Expertise:

Medical Education; ED&I and Professional Identify Formation; Mixed Methods Research

I confirm that I have read this submission and believe that I have an appropriate level of expertise to state that I do not consider it to be of an acceptable scientific standard, for reasons outlined above.

# Reviewer report: Reviewer response for version 1

**DOI:** 10.21956/mep.21293.r36620

**Published:** 2024-08-13

**Authors:** Dan Furmedge

**Affiliations:** 1 Department of Ageing and Health, Guy's and St Thomas' NHS Foundation Trust, London,, UK

This is an excellent paper, well written covering an important topic. 

 The study is well designed, though being able to see the SBAs which I couldn't access would have been useful. I also wondered why there was not a group that included all of the various demographics i.e. trialled providing every stem with the standard demographics vs none vs some that had some but not others.

 Otherwise, I think this is a very good paper, with relevant and interesting discussion and paves the way for a larger study into this area. If further work in this area is considered it may be worth involving Chris McManus or Kath Woolf to conduct a larger study which can perhaps answer some of the questions that remain.

Have any limitations of the research been acknowledged?

Yes

Is the work clearly and accurately presented and does it cite the current literature?

Yes

If applicable, is the statistical analysis and its interpretation appropriate?

Yes

Are all the source data underlying the results available to ensure full reproducibility?

Yes

Is the study design appropriate and is the work technically sound?

Yes

Are the conclusions drawn adequately supported by the results?

Yes

Are sufficient details of methods and analysis provided to allow replication by others?

Yes

Reviewer Expertise:

NA

I confirm that I have read this submission and believe that I have an appropriate level of expertise to confirm that it is of an acceptable scientific standard.
